# Transcriptome analysis of resistant and susceptible mulberry responses to *Meloidogyne enterolobii* infection

**DOI:** 10.1186/s12870-021-03128-w

**Published:** 2021-07-16

**Authors:** Hudie Shao, Yu Fu, Pan Zhang, Chunping You, Chuanren Li, Huan Peng

**Affiliations:** 1 College of Agriculture, Yangtze University, Jingzhou, 434025 Hubei China; 2 The Innovative Institute for Plant Health, Zhongkai University of Agriculture and Engineering, Guangzhou, 510225 Guangdong China; 3 StateKey Laboratory for Biology of Plant Diseases and Insect Pests, Institute of Plant Protection, Chinese Academy of Agricultural Sciences, Beijing, 100193 P. R. China

**Keywords:** Mulberry, Sericulture, *Meloidogyne enterolobii*, Resistance evaluation, Transcriptome, Resistance mechanism

## Abstract

**Background:**

Mulberry (*Morus alba* L.) is an important sericulture crop; however, root-knot nematode infection seriously limits its production. Understanding the mechanism of interaction between mulberry and nematode is important for control of infection.

**Results:**

Using sequencing and de novo transcriptome assembly, we identified 55,894 unigenes from root samples of resistant and susceptible mulberry cultivars at different stages after infection with the nematode *Meloidogyne enterolobii*; 33,987 of these were annotated in the Nr, SWISS-PROT, KEGG, and KOG databases. Gene ontology and pathway enrichment analyses of differentially expressed genes (DEGs) revealed key genes involved in hormone metabolic processes, plant hormone signal transduction, flavonoid biosynthesis, phenylpropanoid biosynthesis, and peroxisomal and photosynthetic pathways. Analysis of key trends in co-expression networks indicated that expression of unigenes 0,015,083, 0,073,272, 0,004,006, and 0,000,628 was positively correlated with resistance to *M. enterolobii*. Unigene 0015083 encodes tabersonine 16-O-methyltransferase (16OMT), which is involved in alkaloid biosynthesis. Unigene 0073272 encodes a transcription factor contributing to nitric oxide accumulation during plant immune responses. Unigenes 0,004,006 and 0,000,628 encode ERF and MYB transcription factors, respectively, involved in plant hormone signaling. We verified the accuracy of transcriptome sequencing results by RT-qPCR of 21 DEGs.

**Conclusions:**

The results of this study increase our understanding of the resistance mechanisms and candidate genes involved in mulberry–*M. enterolobii* interaction. Thus, our data will contribute to the development of effective control measures against this pathogen.

**Supplementary Information:**

The online version contains supplementary material available at 10.1186/s12870-021-03128-w.

## Background

Mulberry (*Morus alba* L.) is an economically important sericulture crop used not only for sericulture and silk weaving, but also for healthcare products, medicine, edible fungus cultivation, and in other high value-added fields [1]. Development of the sericulture industry has resulted in the occurrence of several pests and diseases in rapid succession, including root-knot nematode disease, an important disease occurring in mulberry plantations across China [2]. Using morphological and molecular identification methods, Zhang et al. [3] identified the pathogen causing mulberry root-knot nematode disease in Guangdong Province as *Meloidogyne enterolobii*. This root-knot nematode causes serious damage to economic and food crops [4] and has become one of the most threatening pathogenic nematodes in both tropical and subtropical regions of the world, causing estimated potential yield losses of 20% [5]. *M. enterolobii* was first discovered on *Euterolobium contortisiliquum* in Zhangzhou, Hainan Province, China [6]. It can parasitize a variety of economically important crops and tropical fruits and reproduces rapidly. Furthermore, *M. enterolobii* can overcome the resistance response mediated by Mi-resistant nematode genes, resulting in more than 65% yield loss in resistant *Solanum tuberosum*, chili-pepper (*Capsicum* spp.), and other vegetables [5]. *M. enterolobii* has also been found in Africa, America, and Europe [7], with the European and Mediterranean Plant Protection Organization (EPPO) including this species in the EPPO A2 lists in 2010 [8]. To date, *M. enterolobii* has been detected in Hainan, Guangdong, Fujian, Hunan, and Yunnan, among other regions of China [9–13], with a gradual northward spread noted [14].

During their long evolution, plants have formed defense systems against invading root-knot nematodes. The first line of defense is established by extracellular immune receptors that recognize pathogen-associated molecular patterns (PAMPs) corresponding to various pathogens [15]. The immune response caused by PAMP monitoring is referred to as PAMP-triggered immunity (PTI). Molecules triggering PTI responses, such as Flg22, induce expression of PTI response-related genes, which cause reactive oxygen species bursts, stomata closure, and callose accumulation [16]. Nevertheless, pathogens have evolved mechanisms to overcome this first line of basic plant immune defense and can suppress plant immunity by secreting effectors, which may directly inhibit plant immunity-related proteins or change their active state [15]. Hence, various plant regulatory factors work in concert with each other in a highly controlled regulatory network [17].

Understanding how nematodes interact with mulberry is key for controlling infection. Transcriptome sequencing technology can help reveal crosstalk and integration pathways between plants (resistant/ susceptible) and nematodes [17]. Comparative transcriptome analysis using RNA-seq offers an affordable and high-resolution approach for identifying genes that are differentially expressed in response to diverse biotic stresses and has been used in many model and non-model species [17]. Haegeman et al. [18] sequenced *Meloidogyne graminicola* on the Illumina 454 sequencing platform, using a de novo sequencing approach. Further, Santini et al. [19] studied the host transcription spectrum during the affinity interaction between *Phaseolus vulgaris* and *Meloidogyne incognita*, identifying 797 host genes as differentially expressed. Meanwhile, Shukla et al. [20] studied the interaction of susceptible and resistant tomato (*Solanum lycopersicum*) varieties with *M. incognita* using transcriptome analysis.

Although RNA-seq has been used successfully for identifying nematode–host interactions in several different hosts [18], no study has yet been conducted to understand nematode–host interactions in mulberry plants. Paestakahashi et al. [21] were the first to report parasitic damage caused by *M. enterolobii* on mulberry in Brazil. Subsequent morphological and molecular analyses conducted by Zhang et al. [3] identified *M. enterolobii* as the pathogen responsible for mulberry infections in Guangdong Province. Shao et al. [22] examined the resistance of 19 mulberry varieties in Guangdong Province, demonstrating that mulberry C44 is resistant to *M. enterolobii* while 283 × anti^−10^ is a susceptible variety. There is an important need, therefore, to analyze the interactions between resistant and susceptible mulberry varieties and *M enterolobii* using RNA-seq.

In this investigation, we analyzed transcriptome data from disease-resistant and -susceptible varieties of mulberry infected by root-knot nematodes to identify genes and pathways that might be used to develop effective control measures against this pathogen.

## Results

### Transcriptome sequencing

Root samples of the resistant variety Yuesang C44 were collected at different time points and numbered as follows: before inoculation, KB0d; at 8 days post-inoculation (dpi), KBjz8d and KBck8d; at 17 dpi, KBjz17d and KBck17d; and at 23 dpi, KBjz23d and KBck23d; samples labeled with the letters ‘jz’ were inoculated with nematodes while those labeled with ‘ck’ were controls inoculated with water. Similarly, samples of the susceptible variety 283 × anti^−10^ were numbered GB0d, GBjz8d, GBck8d, GBjz17d, GBck17d, GBjz23d, and GBck23d. Technical replicates (samples collected at the same time from the same experimental group) were numbered ①, ②, and ③, giving 42 samples in total. Transcriptome sequencing of the root samples from resistant and susceptible varieties [22] infected with *M. enterolobii* was carried out on the Illumina platform (Table 1). The proportion of high-quality sequences after filtering all reads was > 99%, Q20 (%) was > 98%, and Q30 (%) was > 94%, indicating that the transcriptome data were of good quality and the sequencing results were highly reliable (Table 1).

**Table 1:** Transcriptome sequencing data analysis

Sample	Raw reads, n^a^	Clean reads, n (%)^b^	Q20 (99% base call accuracy), %^c^	Q30 (99.9% base call accuracy), %^d^	GC content, %^e^
KB0d-①	60,909,044	60,493,992 (99.32%)	98.2	94.71	46.43
KB0d-②	62,997,456	62,491,878 (99.2%)	98.68	96.05	45.91
KB0d-③	66,197,912	65,647,714 (99.17%)	98.5	95.33	46.73
GB0d-①	54,258,632	53,912,710 (99.36%)	98.31	94.91	46.89
GB0d-②	64,601,656	64,131,504 (99.27%)	98.78	96.17	46.9
GB0d-③	63,892,702	63,436,502 (99.29%)	98.83	96.35	47.0
KBjz8d-①	59,244,696	58,881,156 (99.39%)	98.33	94.97	47.05
KBjz8d-②	60,152,896	59,777,740 (99.38%)	98.22	94.69	47.23
KBjz8d-③	57,259,132	56,906,180 (99.38%)	98.31	94.91	47.33
KBck8d-①	60,755,908	60,391,936 (99.4%)	98.32	94.92	47.74
KBck8d-②	52,040,558	51,686,572 (99.32%)	98.26	94.81	47.29
KBck8d-③	64,174,538	63,719,678 (99.29%)	98.27	94.82	47.34
GBjz8d-①	68,430,676	67,917,050 (99.25%)	98.22	94.72	47.96
GBjz8d-②	63,719,816	63,292,076 (99.33%)	98.26	94.81	47.32
GBjz8d-③	61,292,316	60,748,664 (99.11%)	98.22	94.77	46.92
GBck8d-①	48,996,092	48,647,074 (99.29%)	98.28	94.88	47.62
GBck8d-②	85,457,146	84,877,082 (99.32%)	98.29	94.9	48.08
GBck8d-③	63,423,860	62,959,404 (99.27%)	98.16	94.6	46.78
KBjz17d-①	81,179,024	80,436,262 (99.09%)	98.2	94.69	47.1
KBjz17d-②	64,554,492	64,163,368 (99.39%)	98.33	94.93	47.82
KBjz17d-③	50,325,862	50,006,746 (99.37%)	98.32	94.91	47.69
KBck17d-①	57,315,338	56,890,984 (99.26%)	98.25	94.83	48.96
KBck17d-②	58,278,390	57,858,956 (99.28%)	98.32	95.01	49.78
KBck17d-③	60,404,646	59,916,916 (99.19%)	98.85	96.37	48.53
GBjz17d-①	60,396,566	59,987,776 (99.32%)	98.4	95.15	47.6
GBjz17d-②	73,025,484	72,582,168 (99.39%)	98.33	94.98	47.74
GBjz17d-③	72,193,056	71,733,542 (99.36%)	98.2	94.63	47.68
GBck17d-①	54,270,666	53,902,126 (99.32%)	98.44	95.29	46.68
GBck17d-②	64,310,188	63,735,094 (99.11%)	98.72	96.18	46.82
GBck17d-③	55,197,462	54,697,628 (99.09%)	98.69	96.14	47.8
KBjz23d-①	59,095,200	58,691,120 (99.32%)	98.31	94.93	47.14
KBjz23d-②	79,210,406	78,762,440 (99.43%)	98.33	94.96	47.72
KBjz23d-③	61,962,352	61,612,726 (99.44%)	98.41	95.16	47.66
KBck23d-①	57,595,244	57,045,606 (99.05%)	98.78	96.32	49.15
KBck23d-②	63,076,062	62,601,422 (99.25%)	98.77	96.3	47.09
KBck23d-③	63,769,422	63,342,658 (99.33%)	98.83	96.38	47.32
GBjz23d-①	64,686,018	64,110,442 (99.11%)	98.24	94.82	47.08
GBjz23d-②	69,797,772	69,378,514 (99.4%)	98.32	94.94	48.46
GBjz23d-③	47,339,138	47,004,460 (99.29%)	98.22	94.75	49.18
GBck23d-①	52,537,608	52,003,500 (98.98%)	98.72	96.21	49.64
GBck23d-②	60,610,656	60,072,490 (99.11%)	98.65	95.94	47.82
GBck23d-③	63,395,176	62,774,902 (99.02%)	98.71	96.13	48.32

^a^Number of original data reads

^b^Number of high-quality sequences (and percentage) of raw reads after filtering

^c^Percentage of bases with an accuracy of ≥ 99% after filtering

^d^Percentage of bases with an accuracy of ≥ 99.9% after filtering

^e^Percentage of G/C nucleotides in the sequence

### Results of de novo assembly

**Fig. 1:**
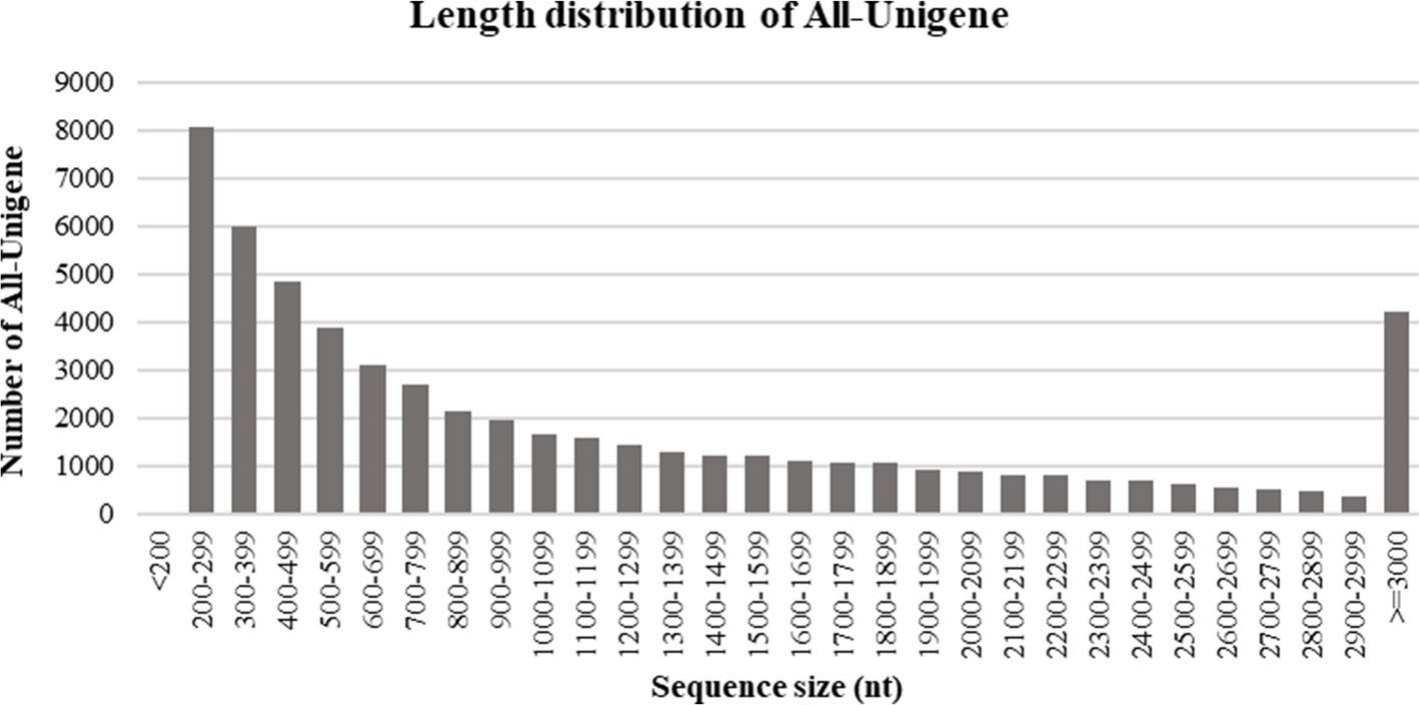
Transcript length distribution

We used Trinity software to assemble the clean reads. After assembly, 55,894 unigenes were obtained, with a length range of 201–22,885 bp (mean, 1,202 bp; N50, 1,964 bp) and a mean GC content of 41.09%. The assembled transcript length distribution is illustrated in Fig. 1.

### Unigene function annotation

We used BLAST X to compare the assembled unigenes with protein sequences in the databases Nr, SWISS-PROT, KEGG, and KOG (E value < 0.00001) for annotation of protein function. A total of 33,987 unigenes were annotated, of which 33,921 were annotated in the Nr database, accounting for 99.8% of all annotated genes; 20,750 were annotated in the SWISS-PROT database, representing 61.1% of all annotated genes; 13,165 were annotated in the KEGG database, representing 38.7% of all annotated genes; and 17,462 were annotated in the KOG database, accounting for 51.4% of all annotated genes.

### Screening for differentially expressed genes (DEGs)

**Fig. 2:**
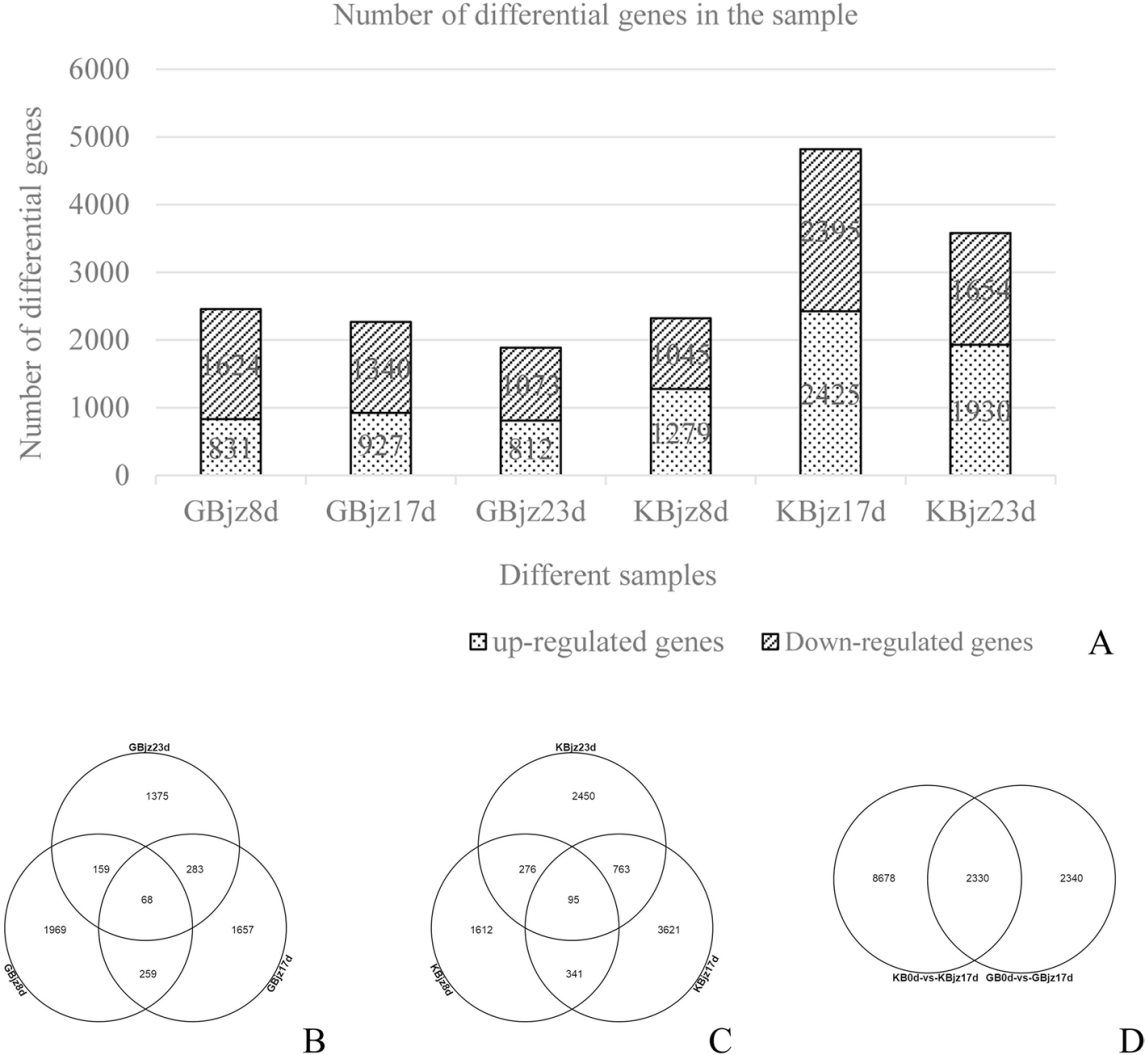
DEGs in mulberry inoculated with root-knot nematodes. **a** Number of DEGs in each sample relative to control samples before inoculation. **b** Venn diagram comparing DEGs in inoculated roots versus uninoculated (control) roots of 283 × anti^−10^ (susceptible variety) at different time points. **c** Venn diagram comparing DEGs in inoculated roots versus uninoculated (control) roots of Yuesang C44 (resistant variety) at different time points. **d** Venn diagram comparing DEGS in 283 × anti^−10^ and Yuesang C44 at 17 days after inoculation

DEGs in resistant and susceptible varieties at different time points were identified using the threshold p < 0.01 and fold-change (FC) ≥ 2 (Fig. 2a). A Venn diagram comparing DEGs in inoculated roots versus uninoculated (control) roots at the three time points revealed 2,455, 2,267, and 1,885 DEGs in the susceptible cultivar at 8, 17 and 23 dpi, respectively, among which 1,969, 1,657, and 1,375 DEGs were unique to each time point (Fig. 2b). In the resistant variety, 2,324, 4,820, and 3,584 DEGs were identified at 8, 17, and 23 dpi, respectively, among which 1,612, 3,621, and 2,450 were unique to each time point (Fig. 2c).

We next compared gene expression in resistant and susceptible varieties at 17 dpi, relative to that before inoculation, using a Venn diagram of DEGs for gb0d-vs-gbjz17d and kb0d-vs-kbjz17d (representing DEGs in GBjz17d and KBjz17d, respectively, relative to expression levels at GB0d and KB0d). Figure 2d shows that 8,678 DEGs were unique to the resistant variety at 17 dpi while 2,340 were unique to the susceptible variety and 2,330 were common to both varieties.

### Gene Ontology (GO) enrichment analysis of DEGs

#### GO enrichment analysis of DEGs in susceptible mulberry at 17 dpi

**Fig. 3:**
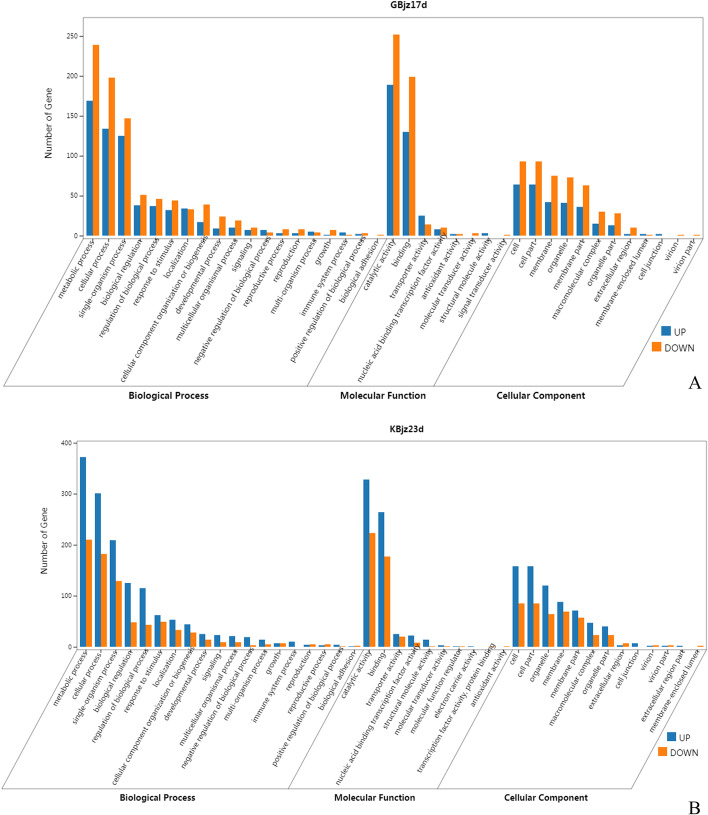
GO classification of DEGs in mulberry after inoculation with nematodes. DEGs in susceptible mulberry at 17 dpi (GBjz17d) (**a**) and in resistant mulberry at 23 dpi (KBjz23d) (**b**), relative to expression in uninoculated (control) samples

Comparing the 2,267 DEGs associated with nematode inoculation in the susceptible mulberry cultivar at 17 dpi (GBjz17d) with the GO database (Fig. 3a) indicated that these genes were enriched for 1,129 GO entries. There were 747, 280, and 102 GO items in the three major ontologies of biological process, molecular function, and cell component, respectively. The number of down-regulated genes associated with each item was statistical significance higher than the number of up-regulated genes. In the biological process ontology, the greatest proportions of genes were in the classifications metabolic process, cellular process, and single-organism process. In the ontology of molecular function, most of the genes were in the catalytic activity and protein binding classifications. In the cell component ontology, large numbers of genes were associated with the cell, cell part, membrane, organelle, and membrane part classifications (Fig. 3a).

#### GO enrichment analysis of DEGs in resistant mulberry at 23 dpi

Comparing the 3,584 DEGs associated with nematode inoculation in the resistant mulberry cultivar at 23 dpi (KBjz23d) with the GO database (Fig. 3b) demonstrated that these genes were enriched for 1,309 GO entries, including 898, 292, and 119 GO entries in the three major ontologies of biological process, molecular function, and cell composition, respectively. The number of up-regulated genes associated with each entry was significantly higher than the number of down-regulated genes. In the three ontologies, the GO classifications of enriched DEGs for KBjz23d were the same as those for GBjz17d; however, the enriched GO entries differed from those for GBjz17d; in the biological process ontology, KBjz23d DEGs were significantly enriched for entries related to the regulation of macromolecular metabolism, while GBjz17d DEGs were significantly enriched for entries related to plant cell wall metabolism.

#### GO enrichment analysis of DEGs between resistant and susceptible varieties

**Fig. 4:**
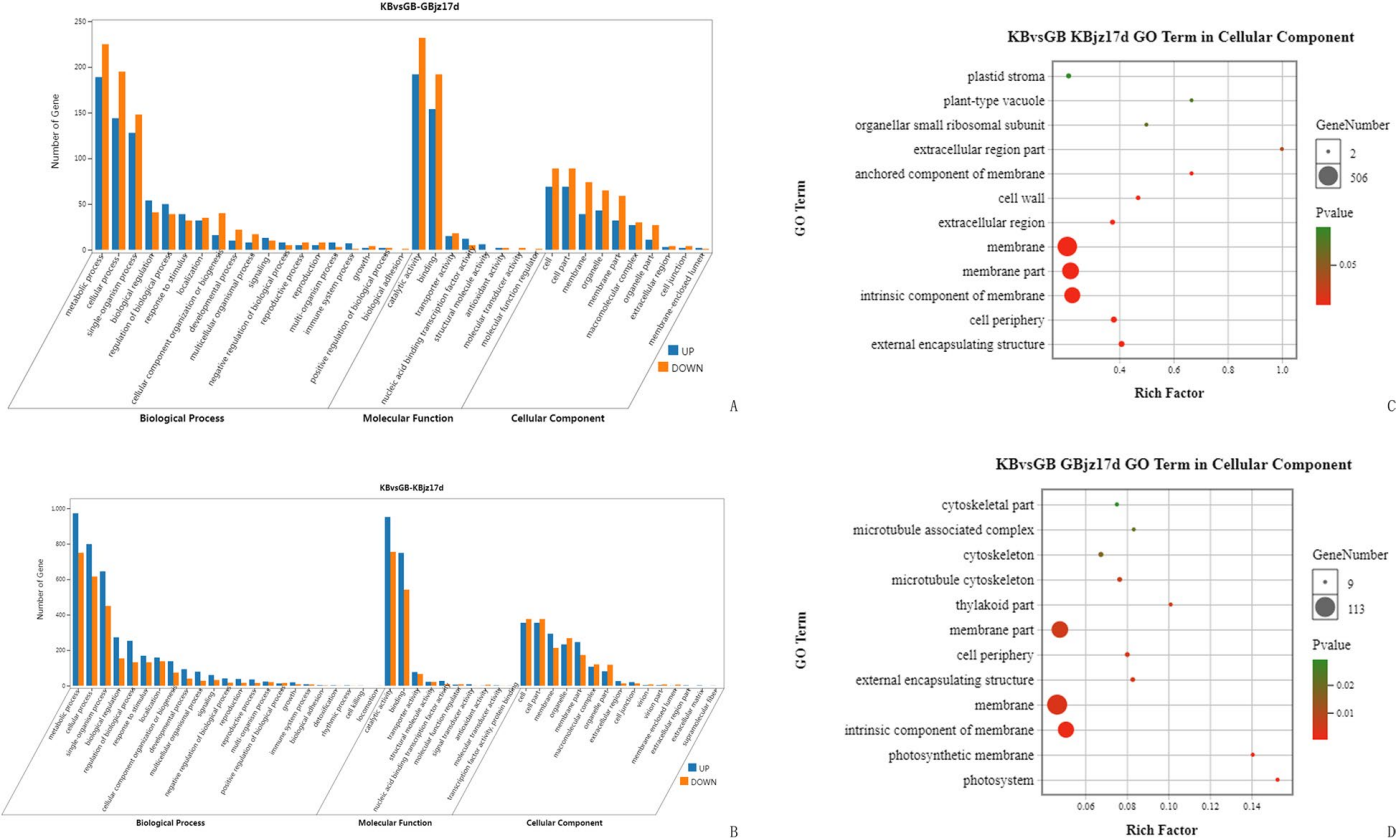
GO classification and GO enrichment results of DEGs in resistant and susceptible varieties relative to expression before inoculation. (**a)** GO classification of DEGs in susceptible mulberry at 17 days after inoculation with nematodes (GBjz17d). (**b)** GO classification of DEGs in resistant mulberry at 23 days after inoculation with nematodes (KBjz23d). (**c)** GO enrichment results of DEGs in KBjz23d. (**d)** GO enrichment results of DEGs in GBjz17d

Comparing the DEGs identified in resistant and susceptible varieties at 17 dpi relative to expression before inoculation, we identified 2,340 DEGs (referred to as KBvsGB-GBjz17d DEGs) unique to the susceptible variety (Fig. 2d). The GO database showed that these DEGs were mainly enriched for 1,146 GO items (Fig. 4a), with 776, 258, and 112 in the ontologies biological process, molecular function, and cell composition, respectively. The number of genes down-regulated in the susceptible variety was significantly higher than the number of genes up-regulated. In the biological process ontology, the highest proportions of genes were in the classifications metabolic process, cellular process, and single organization process, while for the molecular function ontology, the largest numbers of genes were associated with the classification’s catalytic activity and protein binding; in the cell component ontology, the classification cells and cell parts encompassed the greatest percentage of genes (Fig. 4a).

We next analyzed the 8,678 DEGs at 17 dpi relative to expression before inoculation that were unique to the resistant variety (KBvsGB-KBjz17d DEGs; Fig. 2d). The GO database suggested (Fig. 4b) that KBvsGB-KBjz17d DEGs were mainly enriched for 1,764 GO entries, among which there were 1,179, 422, and 163 GO entries in the three major ontologies biological process, molecular function, and cell composition, respectively. The number of down-regulated genes associated with each entry was significantly higher than the number of up-regulated genes. In the three ontologies, the GO classifications enriched among KBvsGB-KBjz17d DEGs were the same as those enriched among KBvsGB-GBjz17d (Fig. 4), while the enriched GO entries differed from those of KBvsGB-GBjz17d. In the cell component ontology, KBvsGB-KBjz17d DEGs were significantly enriched for items including ribosomes and cell–matrix (Fig. 4c), while those of KBvsGB-GBjz17d were significantly enriched for items related to photosynthesis, such as thylakoid membranes (Fig. 4d).

### Pathway enrichment analysis of DEGs

Pathway analysis is helpful for understanding the biological functions of genes and for identifying the involvement of DEGs in key signal transduction and biochemical metabolism pathways.

### Pathway enrichment analysis of DEGs in susceptible mulberry at 17 dpi

**Fig. 5:**
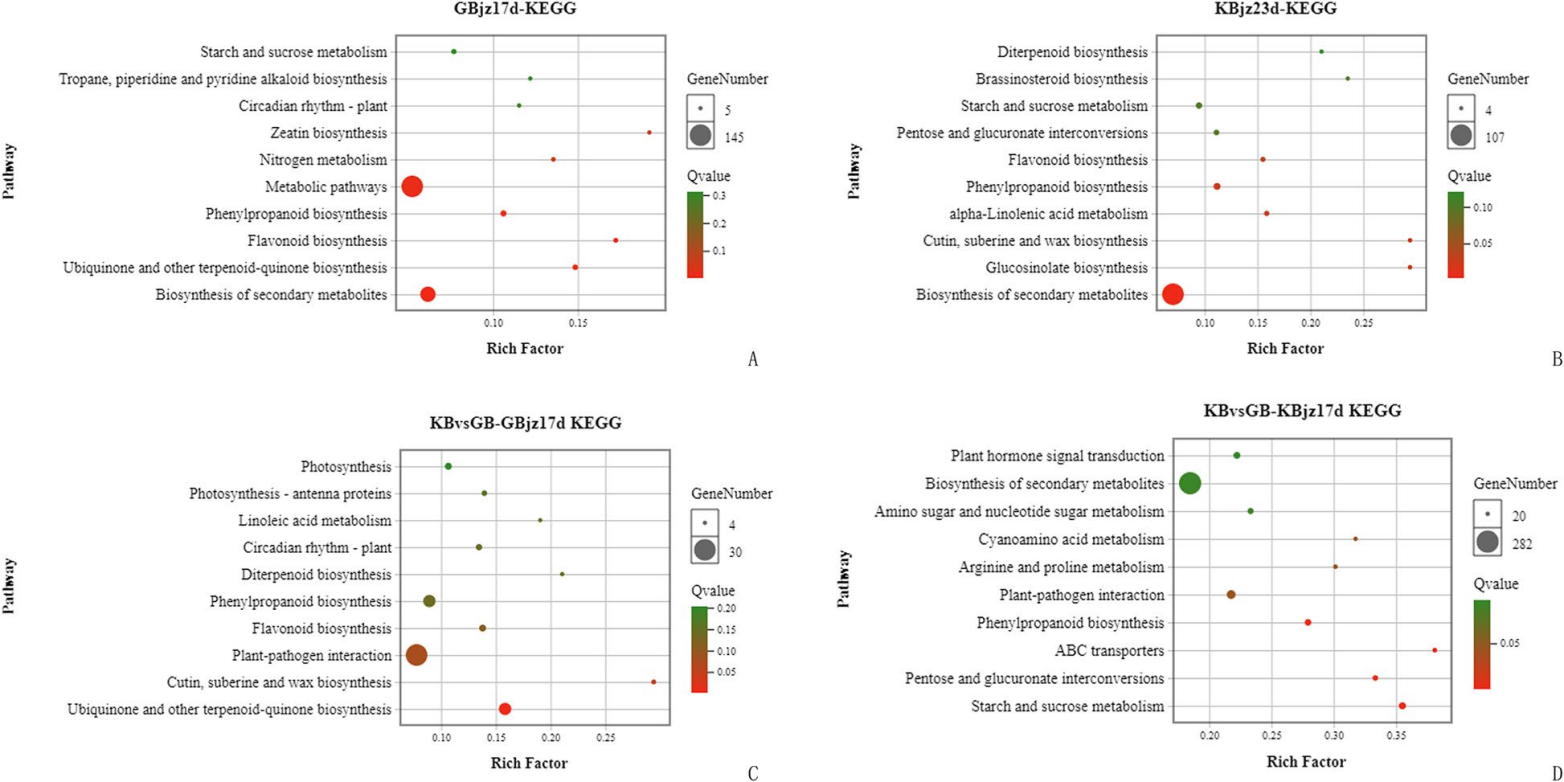
Pathway enrichment results of DEGs in mulberry after inoculation with nematodes. Pathways associated with DEGs in susceptible mulberry at 17 dpi (GBjz17d) (**a**) and resistant mulberry at 23 dpi (KBjz23d) (**b**), relative to expression in uninoculated (control) samples. Pathway enrichment results for DEGs unique to the susceptible (283 × anti^−10^) (**c**), and resistant (Yuesang C44) (**d**) mulberry varieties at 17 dpi, relative to expression before inoculation

Pathway annotation of DEGs in inoculated roots versus uninoculated (control) roots of susceptible mulberry at 17 dpi (GBjz17d) using the KEGG database showed that these DEGs were enriched in 113 pathways, with five significantly enriched pathways (Q ≤ 0.05) (Fig. 5a), as follows: biosynthesis of secondary metabolites; ubiquinone and other terpenoid-quinone biosynthesis; flavonoid biosynthesis; phenylpropanoid biosynthesis; and metabolic pathways. Among them, metabolic pathways had the highest number of enriched genes, with 145 DEGs. The enrichment factor (rich factor) value of the flavonoid biosynthesis pathway was the highest, indicating that this pathway was the most enriched.

### Pathway enrichment analysis of DEGs in resistant mulberry at 23 dpi

DEGs from inoculated roots versus uninoculated (control) roots of resistant mulberry at 23 dpi (KBjz23d) were annotated to 109 pathways using the KEGG database. Six of these pathways were significantly enriched (Q ≤ 0.05) with DEGs (Fig. 5b): biosynthesis of secondary metabolites; glucosinolate biosynthesis; cutin, suberine, and wax biosynthesis; alpha-linolenic acid metabolism; phenylpropanoid biosynthesis; and flavonoid biosynthesis. The secondary metabolite biosynthesis pathway was enriched with the most genes (107 DEGs). The glucosinolate biosynthesis and cutin, suberine, and wax biosynthesis pathways showed the highest levels of enrichment.

### Pathway enrichment analysis of DEGs in resistant and susceptible varieties

DEGs identified in resistant and susceptible mulberry varieties at 17 dpi relative to expression before inoculation were annotated for pathway enrichment using the KEGG database. DEGs unique to the susceptible variety (KBvsGB-GBjz17d) were enriched in 111 pathways, with two pathways significantly enriched (Q ≤ 0.05) (Fig. 5c): ubiquinone and other terpenoid-quinone biosynthesis and cutin, suberine and wax biosynthesis. Cutin, suberine and wax biosynthesis was the most strongly enriched. DEGs unique to resistant mulberry (KBvsGB-KBjz17d) were enriched in 126 pathways, with seven pathways significantly enriched (Q ≤ 0.05) (Fig. 5d), as follows: starch and sucrose metabolism; pentose and glucuronate conversion interconversions; ABC transporters; phenylpropanoid biosynthesis; plant-pathogen interaction; arginine and proline metabolism; and cyan amino acid metabolism. Among these pathways, ABC transporters was the most enriched.

### DEG expression trends

We analyzed the expression trends of DEGs in susceptible mulberry at 17 dpi relative to before inoculation (GBjz17d), DEGs in resistant mulberry at 23 dpi relative to before inoculation (KBjz23d), DEGs at 17 dpi relative to expression before inoculation that were unique to susceptible mulberry (KBvsGB-GBjz17d), and DEGs at 17 dpi relative to expression before inoculation that were unique to resistant mulberry (KBvsGB-KBjz17d). Our analysis resulted in identification of 20 trends (Additional File 1: Figure S1).

### GBjz17d DEG expression trends

Trend analysis of GBjz17d DEGs identified profile2, profile1, profile18, profile16, profile17, and profile3 (Additional File 1: Figure S1a) as significantly enriched trends, representing 452, 312, 210, 219, 196, and 169 genes, respectively.

### KBjz23d DEG expression trends

Trend analysis of KBjz23d DEGs identified profile17, profile2, profile19, profile0, profile12, and profile15 (Additional File 1: Figure S1b) as significantly enriched trends, representing 627, 260, 349, 333, 260, and 342 genes, respectively.

### Expression trends of DEGs between resistant and susceptible varieties

Trend analysis of KBvsGB-GBjz17d DEGs (Additional File 1: Figure S1c) revealed profile2, profile17, profile1, profile16, profile18, profile7, and profile0 as significantly enriched, representing 461, 347, 244, 307, 227, 136, and 106 genes, respectively.

Trend analysis of KBvsGB-KBjz17d DEGs (Additional File 1: Figure S1d) showed that profile2, profile1, profile17, profile18, profile12, profile3, profile7, and profile0 were enriched, representing 2,003, 953, 1,373, 492, 346, 423, 294, and 306 genes, respectively.

### Co-expression network analysis of key trends

The core gene with the highest connectivity in GBjz17d profile1 was unigene 0015188; the co-expression network diagram for this gene is presented in Additional File 1: Figure S2a. Unigene 0015188 encodes β-galactosidase, which is involved in the degradation of pectin in plant primary cell walls. The core gene with the highest connectivity in GBjz17d profile3 was unigene 0022838, and the related co-expression network is presented in Additional File 1: Figure S2b. Unigene 0022838 encodes CASP like protein 1C1, which is related to plant stress resistance. Core genes with high connectivity in GBjz17d profile16 were unigenes 0,022,247 and 0,010,321; the related co-expression network is shown in Additional File 1: Figure S2c. Unigene 0022247 encodes 3-hydroxy-3-methylglutaryl coenzyme A reductase, which is a key enzyme for antitoxin- and steroid-based biosynthesis of sesquiterpenoids. Unigene 0010321 may encode a leucine rich repeat receptor protein kinase (LRR-RLK), which is involved in plant hormone signal transduction, recognition, and transmission between plants and pathogens. The core gene with high connectivity in GBjz17d profile18 was unigene 0021656, and the related co-expression network is detailed in Additional File 1: Figure S2d. Unigene 0021656 encodes calmodulin-dependent protein kinase type I, and KEGG annotation showed that this gene is part of the plant-pathogen interaction pathway.

The core gene with highest connectivity in KBjz23d profile0 was unigene 0014848; the related co-expression network diagram is shown in Additional File 1: Figure S2e. Unigene 0014848 encodes ubiquitin carboxy terminal hydrolase 12, an effector protein that can inhibit plant resistance to root-knot nematodes and promote the formation of nematode feeding points. In KBjz23d profile12, the core gene with highest connectivity was unigene 0015083; the related co-expression network is shown in Additional File 1: Figure S2f. Unigene 0015083 encodes a 16-o-methyltransferase ROMT protein involved in plant alkaloid biosynthesis. The core genes with highest connectivity in the KBjz23d profile15 were unigenes 0,073,558 and 0,073,272, and the related co-expression network is shown in Additional File 1: Figure S2g. Unigene 0073558 encodes ubiquitin carboxyl terminal hydrolase 13, while unigene 0073272 encodes the SRG1 protein, which belongs to the zinc finger transcription factor family. The expression of *srg1* is related to the accumulation of nitric oxide during plant immune responses. Further, GO biological process annotation showed that this gene is related to flavonoid biosynthesis. The core genes with high connectivity in KBjz23d profile19 were unigenes 0,002,889 and 0,068,389, and the related co-expression network diagram is shown in Additional File: Figure S2h. Unigene 0002889 encodes protein phosphatase 2C, which can regulate abscisic acid (ABA) signal transduction, and unigene 00683889 encodes the protein kinase Cipk14, which plays a role in stress responses; GO biological annotation indicated that this gene is related to cell process regulation.

The core gene with highest connectivity in KBvsGB-KBjz17d profile3 was unigene 0004006, and the related co-expression network is shown in Additional File 1: Figure S2i. Unigene 0004006 encodes an ERF transcription factor involved in signal transmission using salicylic acid (SA), ethylene, jasmonic acid, and other factors, which plays an important role in plant stress responses. In KBvsGB-KBjz17d profile12, core genes with high connectivity were unigenes 0,000,628 and 0,015,083; the related co-expression network diagram is presented in Additional File 1: Figure S2j. Unigene 0000628 encodes the MYB transcription factor family protein AIM1, which contributes to stress-related gene expression during ABA signal transmission and positively regulates plant defense responses to pathogens. Unigene 0015083 encodes a rumt glycyrrhizin-16-O-methyltransferase gene, associated with plant alkaloid biosynthesis. The core genes with highest connectivity in KBvsGB-GBjz17d profile16 were unigenes 0,052,595 and 0,004,809, and the related co-expression network is shown in Additional File 1: Figure S2k. Unigene 0052595 encodes the autophagy-related factor Atg8. Unigene 0004809 encodes cell wall-related receptor kinase 22, which belongs to the RLK receptor kinase family involved in plant defense.

### Reverse-transcription quantitative PCR (RT-qPCR) validation of DEGs

**Fig. 6:**
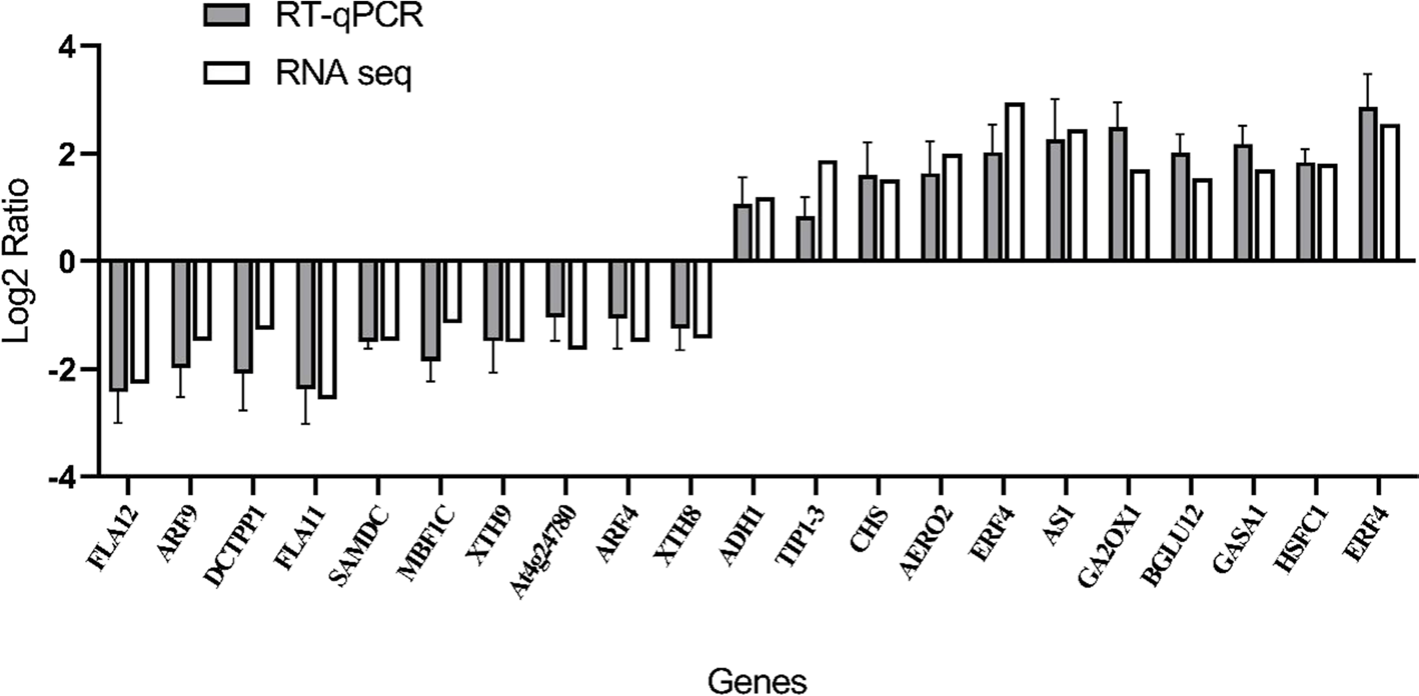
Validation of DEGs by RT-qPCR

To verify the accuracy of the transcriptome sequencing results, we analyzed 21 DEGs by RT-qPCR using cDNA obtained by reverse transcription of the RNA used for sequencing as template. Relative gene expression levels were calculated using the 2^−ΔΔCt^ method, and GraphPad Prism 8.0 used to visualize the results (Fig. 6). Although the magnitude of differences in expression detected by RT-qPCR were not identical to those of DEGs detected by transcriptome sequencing, the direction of the change in DEG expression was consistent using the two approaches, indicating that the results of transcriptome sequencing were highly reliable.

## Discussion

*M. enterolobii* is a mulberry root-knot pathogen [23]. This species displays strong virulence and rapid reproduction; thus, its impact on mulberry production is high. Cultivation of mulberry varieties resistant to *M. enterolobii* is one of the most effective control methods [22]; however, the mechanism underlying resistance of mulberry to *M. enterolobii* is not clear. In this study, we used transcriptome sequencing technology for the first time to our knowledge for studying *M. enterolobii* infection of mulberry, with the aim of understanding the interactions between resistant and susceptible mulberry varieties and *M. enterolobii.*

We selected 21 DEGs related to infection of *M. enterolobii* for RT-qPCR verification. Our results revealed the same trend in DEG expression as established by transcriptome sequencing results, indicating high reliability of the transcriptome sequencing results in reflecting the true expression levels of genes in the roots of mulberry infected by *M. enterolobii*. Transcriptome sequencing indicated that hormone metabolic processes, plant hormone signal transduction, flavonoid biosynthesis, phenylpropanoid biosynthesis, and transport-related genes may be involved in the interaction between *M. enterolobii* and mulberry. Research on the underlying mechanism of interaction will provide further new insights.

At each time point after inoculation with root-knot nematodes, the number of genes down-regulated in the susceptible variety was greater than the number of genes up-regulated, compared with expression in uninoculated controls, with the number of DEGs highest at 8 dpi. These results indicate that the primary plant defense response gradually stabilizes during the mid and late stages of infection (17 and 23 dpi), leading to a decrease in the number of DEGs (Fig. 3). The number of DEGs identified in the resistant variety at 8, 17, and 23 dpi with root-knot nematodes compared with expression in uninoculated controls first increased and then decreased slightly, with the number of up-regulated genes higher than the number of down-regulated genes. These results indicate that the primary defense response of the disease-resistant variety was triggered during the early stage of root-knot nematode infection (8 dpi), while the plant produced a more advanced defense system involving induction of numerous defense-related genes during prolonged infection, which might explain the large increase in the number of DEGs during mid and late infection (17 and 23 dpi). DEGs in resistant and susceptible mulberry varieties infected with *M. enterolobii* were consistent with those of resistant and susceptible watermelon materials infected with *M. incognita*, as reported by Zhao [24]. Bioinformatics analysis of these DEGs showed that they were related to processes involved in nematode resistance, including active oxygen removal, cellulose, lignin, diabetic synthesis, and ABA signaling. In these processes, the expression of many key genes is positively correlated with resistance to *M. incognita*.

We performed GO and pathway enrichment analysis of DEGs at 17 dpi and 23 dpi for susceptible and resistant mulberry varieties, respectively. Four different gene sets were enriched for the same GO classification, and the enriched pathways included secondary metabolite biosynthesis pathways related to plant disease resistance, such as phenylpropane biosynthesis, flavonoid biosynthesis, and quinone biosynthesis. These data show that both resistant and susceptible mulberry varieties deploy similar defense processes after infection. GO enrichment analysis indicated more down-regulated genes than up-regulated genes associated with this GO classification in the susceptible variety, while the opposite was the case (i.e., more up-regulated genes than down-regulated genes) in the disease-resistant variety. Further, the number of DEGs annotated in the GO database was much higher in the resistant variety than that in the susceptible variety. This is consistent with results reported by Xing [25] and shows that resistant varieties can initiate expression of a large number of defense-related genes following infection to improve their resistance to pathogens.

Phenylpropane is a low molecular weight antibiotic, insect repellent, or signaling molecule involved in interactions between plants and microbes through its participation in plant defense responses [26]. The biosynthesis of phenylpropanes is related to the production of disease-resistant secondary biomass, including plant protection factors, lignin, and phenolic compounds, and offers a potential method for improving plant resistance to nematodes [27]. Wuyts et al. [28] studied the lignin and phenylpropane content in roots of resistant and susceptible *Musa nana* Lour infected by *Radopholus similis*, finding that lignin and ferulic acid (phenylpropane) contents were higher in the vascular bundles of resistant varieties than in those of susceptible varieties; all vascular bundles exhibited lignification induced by infection. Xu et al. [29] showed that, after infection with *M. incognita*, phenylpropane content and related enzyme activities in the roots of resistant eggplant varieties were greater than those in susceptible varieties. In the present study, genes related to phenylpropane biosynthesis in susceptible mulberry began to be down-regulated at 17 dpi with nematodes, while those in the resistant variety showed up-regulated expression. This may be one of the reasons for the differences in resistance between resistant and susceptible varieties.

Quinone and flavonoid are phenolic compounds that function as secondary metabolites [30]. Phenolic compounds are important for interactions between plants and pathogens. Dama [31] measured the activity of three types of quinone extracted from plants against *Meloidogyne javanica*. The three substances tested had different degrees of nematode-killing activity, with the killing rate of baihuadan-quinone reaching 100%. Hutangura et al. [32] reported that the formation of giant cells in *Trifolium repens* is related to auxin accumulation, with flavonoids acting as auxin transport regulators, potentially controlling auxin accumulation. Further, flavonoids can inhibit *M. incognita* activity [33], and flavonoid production is a plant defense response to nematode infection [34]. Wasson et al. [35] demonstrated that flavonoids can influence the size and cell division of root-knots, while they are not involved in root-knot formation or mega cell formation and it is unlikely that auxin transport and accumulation are mediated by the root-knot. In this study, we found that genes related to phenolic compound biosynthesis were down-regulated in susceptible mulberry plants in the later stages of infection, while they were up-regulated in resistant varieties.

Infection with root-knot nematodes can influence plant photosynthesis, which is a basic physiological activity of plants required for producing organic matter and storing energy. Loveys and Bird [36] observed that the rate of photosynthesis decreases significantly in the early stage of infection by *M. javanica* compared with controls. Further, Ye et al. [37] studied the effects of nematode infection on the photosynthesis of resistant and susceptible greenhouse-grown cucumber leaves, showing that chlorophyll content and net photosynthetic rate of both resistant and susceptible materials decrease after infection; however, these decreases were significantly smaller in resistant materials than in susceptible materials. Zhao [24] also found that expression of genes related to photosynthesis, chlorophyll binding, the photosynthetic system, and other processes are inhibited during the early stage of *M. incognita* infection of resistant and susceptible watermelon varieties, using transcriptome sequencing technology. In this study, genes related to resistance, photosynthesis, and the thylakoid were inhibited during the early stage of nematode infection, consistent with previous findings.

We detected enrichment of genes related to zeatin biosynthesis and peroxidase in susceptible mulberries. Zeatin is a cytokinin [38], and cytokinin levels are altered when plants are under stress [39]. Lohar et al. [40] showed that expression of cytokinin response regulatory genes decreases when root-knots develop in plant roots, and that overexpression of cytokinin oxidase in *Lotus japonicus* reduces the number of nematode-infected root-knots. In this study, inhibition of zeatin biosynthesis in infected susceptible mulberry varieties may be related to the production of giant cells. Peroxidase is an important plant protective enzyme related to stress resistance [41]. Xu et al. [42] proposed that low peroxidase activity in resistant eggplants infected by root-knot nematodes is beneficial for the accumulation of reactive oxygen species and facilitates allergic necrosis in plant roots. Jin et al. [43] observed the tendency of peroxidase activity to first increase and then decrease during the early stage (0–25 days) of *M. incognita* infection of black seed pumpkin. In this study, the expression of peroxidase-related genes was first up-regulated and then down-regulated after infection of susceptible mulberry. Damage to the root cell membrane of susceptible mulberry cultivars during the early stage of nematode infection may accelerate enzymatic reactions, causing up-regulated expression of related genes; changes in root cells tended to be relatively slow in the later stages of infection, and related genes began to be down-regulated.

In the resistant mulberry variety, genes related to SA metabolism and terpene biosynthesis were also enriched among DEGs and showed up-regulated expression following nematode infection. SA is a simple phenolic compound involved in signal transduction and can induce expression of pathogenicity-related proteins and plant systemic acquired resistance [44]. Further, it is an important signaling molecule causing plant allergic reactions to root-knot nematodes [45]. Terpenes are important secondary metabolites in plants that regulate plant growth and development, help resist photooxidative stress, and directly or indirectly participate in plant defense [46]. Echeverrigaray et al. [47] studied the activity of 22 monoterpene compounds against nematodes, demonstrating that 20 of them could significantly reduce the hatching rate of nematode eggs while 11 could reduce the mobility of root-knot nematodes. In this study, DEGs associated with SA metabolism and the terpenoid pathway were only enriched in the disease-resistant mulberry variety, indicating that the susceptible mulberry variety employs different defense mechanisms from those in the resistant mulberry following infection with root-knot nematodes.

In addition to the enrichment of genes related to SA metabolism and terpenoid biosynthesis among DEGs, some core genes with high connectivity in the resistant mulberry variety co-expression network were zinc-finger, MYB, and ERF transcription factors. Transcription factors can improve plant resistance by regulating transduction of signals involved in plant immune responses to attack by biological and non-biological factors. In this study, we found that expression of genes encoding ERF (unigene 0004006) and MYB (unigene 0000628) transcription factors involved in plant hormone signaling was positively correlated with nematode infection. A large number of plant transcription factor studies have revealed that members of the MYB and ERF transcription factor families can directly or indirectly participate in plant defense responses. Conserved functional domains of these transcription factors interact specifically with cis-elements in the promoter regions of regulatory genes, enabling them to participate in the regulatory network of plant disease resistance-related gene expression [48, 49]. These transcription factors can directly regulate the transcription of plant target genes and interact with other proteins. An activated form is required for participating in signal transduction pathways. We identified unigene 0073272 as a core gene in defense against root-knot nematodes. Unigene 0073272 corresponds to the zinc finger transcription factor *srg1*, whose expression is positively regulated by nitric oxide in plants and can activate plant defense responses [50]. Accumulation of the zinc-finger transcription factor SRG1 is induced following a pathogen-triggered nitro-sative burst. Subsequently, this transcription factor binds to either ACTN6ACT or ACTN4ACT sequences in target genes that presumably include negative regulators of immune function. SRG1 appears to act as a transcriptional repressor, utilizing its putative ERF-associated amphiphilic repression (EAR) domain to recruit the corepressor TOPLESS, contributing to the engagement of plant defense responses and the establishment of immunity [50].

Through co-expression network analysis of key trends, we identified genes positively correlated with resistance to *M. enterolobii*. Unigene 0015083 encodes a tabersonine 16-O-methyltransferase ROMT gene, which is involved in alkaloid biosynthesis. Zhang [49] found that C4 methylsterol oxidase (Mgr005) and sterol C-methyltransferase ROMT (Mgr009) are involved in the biosynthesis of membrane proteins related to the pathogenicity of *Magnaporthe grisea*; They may also be associated with a *Magnaporthe grisea* plasma membrane sodium response protein related to signal transduction during infection. There are few studies on transcription factors encoding genes related to disease resistance in mulberry, and analysis of these genes in follow-up studies will assist in future breeding for nematode resistance in mulberry. By screening for DEGs during the infection process and determining the molecular mechanisms underlying mulberry resistance to root-knot nematode disease, our data provide a theoretical basis for development of disease-resistant mulberry cultivars. In addition, the detailed transcriptome data generated in this study may facilitate identification of genes that can be targeted to increase the resistance of mulberry to *M. enterolobii*.

## Conclusions

We used transcriptome sequencing and de novo assembly to construct reference transcripts of resistant and susceptible mulberry varieties infected by *M. enterolobii*. Selection and enrichment analysis of DEGs, trend analysis of DEGs, and co-expression network analysis of DEGs revealed gene expression patterns of resistant and susceptible mulberry after infection with root-knot nematodes. Our results reveal several reasons for the difference in resistance to *M. enterolobii* between resistant and susceptible mulberry varieties*.* The biosynthesis of secondary metabolites related to plant disease resistance continues to be up-regulated in resistant varieties following infection, while it is inhibited in susceptible varieties. Transcription factor-related genes are highly connected and up-regulated in resistant varieties. Meanwhile, susceptible varieties display recognition and transmission of signals associated with *M. enterolobii* infection and down-regulated expression of genes related to plant defense.

## Methods

### Nematode culture

*M. enterolobii* eggs were isolated from the roots of mulberry plants in the sericulture teaching base at South China Agricultural University (Guang zhou province) and used for inoculation, as described below.

### Plant growth conditions

Yuesang C44 is resistant to *M. enterolobii*, while 283 × anti^−10^ is susceptible [22]. Germination and transplantation were conducted following the method described by Wu [51].

### Nematode collection and inoculation

Nematodes were collected according to the method of Li et al. [52]. Inoculation was performed on 60-day-old mulberry seedlings grown under greenhouse conditions. Soil above the plant roots was first removed to expose the root system. Next, 2 mL of egg suspension was added into the soil near the roots using a pipette. After inoculation, roots were covered with soil. Plants in the control group were inoculated using 2 mL of water. Fifteen pots were inoculated for each treatment. Mulberry seedlings were placed in a greenhouse and watered regularly.

### Collection of mulberry seedling root samples

Preliminary experiments indicated that susceptible mulberry varieties begin to produce root knots at 17 days post-inoculation (dpi) with 500 nematode eggs, while resistant varieties start to produce root knots at 23 dpi. Therefore, samples were collected at four time points as follows: on inoculation (day 0), and at 8, 17, and 23 dpi. At each time point, 10 samples were taken from each experimental group, including control groups, for each of the two varieties. Samples were pooled in groups of three or four to generate three samples for each inoculated or control experimental group at each time point. Root-knot samples were collected according to the method described by Li [53].

### Transcriptome sequencing and data analysis

Samples were sent to Guangzhou Jidio Biotechnology Co., Ltd. for RNA extraction and transcriptome sequencing on the Illumina Hiseq 4000 platform using the PE150 double-ended sequencing approach. Since no complete reference genome information for mulberry is available in the NCBI GenBank, Trinity software was used for de novo genome assembly. Mulberry reference transcripts were obtained on the basis of sequencing results and a non-reference genome. Unigene functional annotation and differential expression analyses were conducted on the resulting sequence assembly, followed by GO function enrichment, pathway function enrichment, and trend analyses of selected DEGs.

Since the susceptible variety began to develop root knots at 17 dpi and the resistant variety started to form root knots at 23 dpi, data analysis focused primarily on these two time points (i.e., 17 and 23 dpi) and on DEGs from infected mulberries of the susceptible variety at 17 dpi (GBjz17d) and the resistant variety at 23 dpi (KBjz23d). When comparing differences between resistant and susceptible varieties after infection, GBjz17d and KBjz17d groups were compared, and the DEGs identified were analyzed.

### Quantitative analysis of genes and screening for DEGs

The reads per kilobase per million reads method [54] was used to measure the expression of unigenes. DEGs were screened using DESeq2, with p < 0.01 and fold-change (FC) ≥ 2 selected as the threshold for significantly differential expression. The experiment comprised one resistant and one susceptible variety, with samples collected at four time points. DEGs showing expression changes due to *M. enterolobii* infection were identified, while DEGs exhibiting expression changes during natural mulberry growth were excluded.

### GO terms and KEGG pathway analysis

DEGs were analyzed using GO and KEGG [55] pathway enrichment methods in DAVID [56], using the parameters p < 0.05, gene number ≥ 2, and false discovery rate < 0.01.

### Series test of cluster analysis of DEGs

EdgR software was used to screen DEGs, with a threshold of greater than two times difference in expression and false discovery rate < 0.05. Series test of cluster analysis was performed using STEM software and log2 standardization to preprocess data. The most representative 20 modules were generated by default, and those with p < 0.05 were considered significantly enriched trends. GO and pathway enrichment analyses were carried out for genes identified in each trend to obtain significantly enriched GO terms and metabolic pathways.

### Gene co-expression network analysis

Co-expression network analysis was conducted on DEGs included in enrichment trends. Correlation of all genes in the trend from samples at each time point was evaluated using the Pearson correlation coefficient, and a network diagram based on relationships with Spearman correlation coefficients > 0.9 was drawn [24]. The soft threshold was used to calculate gene connectivity, arrange genes according to their level of connectivity, and identify core genes in the co-expression network (hub genes, representing those with the highest connectivity).

### Validation of DEGs by reverse-transcription quantitative PCR (RT-qPCR)

Twenty-one DEGs related to infection by *M. enterolobii* were selected for RT-qPCR verification.

### Primer design

Primers for RT-qPCR were designed using the primer design tool from NCBI (https://www.ncbi.nlm.nih.gov/). Primers (17–25 nucleotides; GC content, 45%–55%) were designed to generate PCR products of 80–150 bp, ensuring similar melting temperatures between the primers of each pair and avoiding GC or AT enrichment at the 3' terminus. The *β-actin* gene (GenBank accession no. DQ785808), which is stably expressed in mulberry, was used as a reference [57]. Primer sequences are provided in Table 2. Primer synthesis was carried out by Tianyi Huiyuan Company (Guangzhou, China).

**Table 2:** Primer sequences

Unigene ID	Forward primer	Reverse primer
0,014,355	TCTCCCGTCTCATCGAGGCTTA	ACCAAGCACGTTGGCTCTCTA
0,066,216	TCTGGCCAAGCAGATCAACGA	ATCTTTCCCCTGAGCTTGACGC
0,050,791	GTGCTGTTCTTCGACGCTCTCA	TTTCCTCCCGATCCGAACTCCA
0,065,349	AACGTCGCAAAGGGTTCTCC	TGCCACAAGCCCTATTGCAG
0,062,337	TTTCGGTTGGCGCGAACATC	AGCAAGCAACGACGGAACCA
0,023,187	ACCGCCACAGGCTAATGAAGT	TGGTGTCTGTCCCTTGGCTT
0,047,645	TGGCTGATGTTTGTGGCCTTGA	AGTTGCTCTTTGATTGGTGGTGG
0,026,072	TTCTGCTGATACGGCGTCCT	TAGGGCCGCAGAGCTTGATT
0,002,892	GGCATGGCTACTTCCATGTGGT	ATGGTTGGGTTTGCGCTTCC
0,008,985	AGCTCCATTTGGAACGCGGA	GTCGAATCCCTTGTATGAGGCCA
0,059,588	GCAGCAGATGATGGCCTTTC	TGGCGCTTCTTCTCCTTGAG
0,059,369	CGTCAAGGCCAAGACCAATTTCC	CGCCGACGTTGATGTTGTTCTC
0,017,931	ATTGGAATCCGAAGCCGTGGA	TGTCGCCTTTCCGTCCAATCT
0,004,573	GCATCTCTAGTGGCTGCTGCTT	TGGCCGTCTTCAGAGGTTGT
0,018,547	TTTTCCGCCGGGTCGATGTT	TTGACGGCAGTGGCATGAGA
0,026,157	AGAAGGAGGAGGAAGAGGAAGTGT	TGAGGCACGTTTGCGAAGCA
0,070,844	AGTTCTTCATCCGTCGCTCCA	ACAATCTCGCAGACGGCACA
0,026,640	TGATCCAACGAGGGGGTCAT	GGCTTGAATGAAGAAGTTGCTCGG
0,014,688	AGTGGGCGACCAGATCAACA	TGGTTGAGAGTGCCGGTCTT
0,069,934	TCGACATTTTCGGGAAGGCCA	AGCATCATCGGAGTCGCTGT
0,071,495	GCAATGGGGACTGTGACAACA	AGGCATGCCAAAGCTCCAAG
β-actin	AGCAACTGGGATGACATGGAGA	CGACCACTGGCGTAAAGGGA

### Reverse transcription of RNA samples

Evo M-MLV RT Premix for qPCR (AG11706) from Aikerui Biological Engineering Co., Ltd. (Hunan, China) was used to synthesize cDNA from all RNA samples by reverse transcription (RT).

### Fluorescence quantitative PCR

Experiments were performed on a LightCycler® 480 (Roche) fluorescent quantitative PCR instrument using a SYBR® Green Pro Taq HS qPCR Kit from Ecory Bioengineering Co., Ltd (Hunan, China). The qPCR reaction solution system is shown in Table S1. All reagents except the cDNA template were added to a 1.5 mL centrifuge tube, mixed, and then aliquoted into eight-tube strips dedicated for fluorescence quantification. The corresponding cDNA was then added to the eight-tube strip. Experiments were repeated three times, with three negative controls for each pair of primers (without cDNA template). Table S2 shows fluorescence quantitative reaction conditions.

## Supplementary Information

**Additional file 1: Fig. S1.** DEG expression trends. a DEGs in susceptible mulberry at 17 days after inoculation with nematodes relative to expression in uninoculated (control) samples (GBjz17d). b DEGs in resistant mulberry at 23 days after inoculation with nematodes relative to expression in uninoculated (control) samples (KBjz23d). c DEGs at 17 dpi relative to expression before inoculation that were unique to susceptible mulberry (KBvsGB-GBjz17d). d DEGs at 17 dpi relative to expression before inoculation that were unique to resistant mulberry (KBvsGB-KBjz17d). The number of genes for each profile is also shown. The lower left corner of each trend block shows the P-value. Smaller values indicate stronger enrichment (as shown in the graph below). Colors indicate significantly enriched trends, while those blocks without color show non-significantly enriched trends. **Fig. S2.** Co-expression network analysis diagrams showing key trends. a GBjz17d profile1. b GBjz17d profile3. c GBjz17d profile16. d GBjz17d profile18. e KBjz23d profile0. f KBjz23d profile12. g KBjz23d profile15. h KBjz23d profile19. i KBvsGB-KBjz17d profile3. j KBvsGB-KBjz17d profile12. k KBvsGB-GBjz17d profile16.

**Additional file 2: Table S1.** qPCR reaction system. **Table S2.** Conditions for qPCR reaction.

## Data Availability

The assembled gene sequences of *M. enterolobii* are available from NCBI. The RNA-Seq data of *M. enterolobii* are available from NCBI SRA database. Database link is: https://www.ncbi.nlm.nih.gov/biosample, BioSample ID: SAMN18837484 – SAMN18837525. The authors affirm that all data necessary for confirming the conclusions of the article are present within the article, figures, and tables.

## Abbreviations

ABA: Abscisic acid
DEG: Differentially expressed gene
dpi: Days post-inoculation
EPPO: European and Mediterranean Plant Protection Organization
PAMP: Pathogen-associated molecular pattern
PTI: PAMP-triggered immunity
RT: Reverse transcription
SA: Salicylic acid
