# Supplementary material for: Transcriptome analysis of resistant and susceptible mulberry responses to Meloidogyne enterolobii infection
Source: BMC Plant Biol. 2021 Jul 16;21:338. doi: 10.1186/s12870-021-03128-w (PMC8285880; doi:10.1186/s12870-021-03128-w)
Supplement: Supplementary file 1 — Additional file 1: Fig. S1. DEG expression trends. a DEGs in susceptible mulberry at 17 days after inoculation with nematodes relative to expression in uninoculated (control) samples (GBjz17d). b DEGs in resistant mulberry at 23 days after inoculation with nematodes relative to expression in uninoculated (control) samples (KBjz23d). c DEGs at 17 dpi relative to expression before inoculation that were unique to susceptible mulberry (KBvsGB-GBjz17d). d DEGs at 17 dpi relative to expression before inoculation that were unique to resistant mulberry (KBvsGB-KBjz17d). The number of genes for each profile is also shown. The lower left corner of each trend block shows the P-value. Smaller values indicate stronger enrichment (as shown in the graph below). Colors indicate significantly enriched trends, while those blocks without color show non-significantly enriched trends. Fig. S2. Co-expression network analysis diagrams showing key trends. a GBjz17d profile1. b GBjz17d profile3. c GBjz17d profile16. d GBjz17d profile18. e KBjz23d profile0. f KBjz23d profile12. g KBjz23d profile15. h KBjz23d profile19. i KBvsGB-KBjz17d profile3. j KBvsGB-KBjz17d profile12. k KBvsGB-GBjz17d profile16. [file 12870_2021_3128_MOESM1_ESM.pdf]

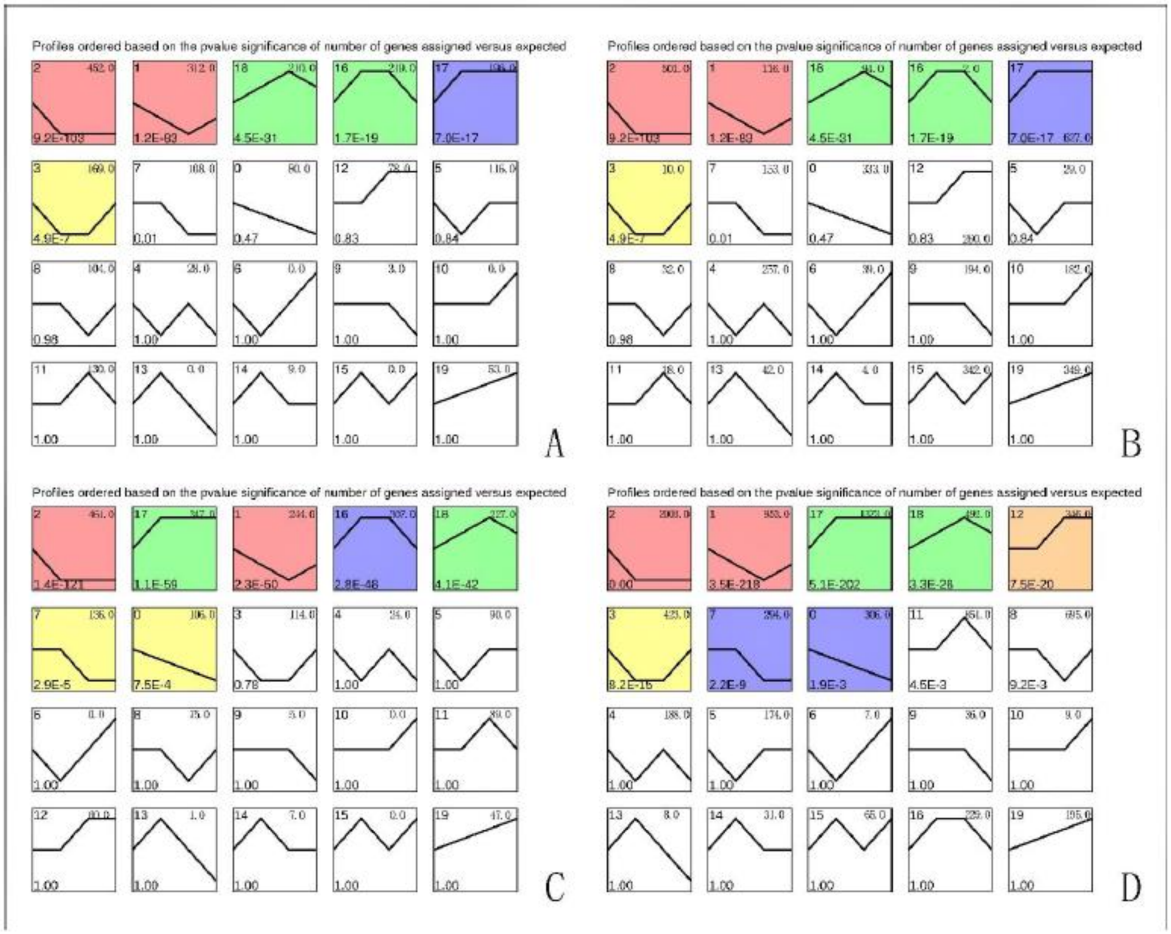

Fig. S1 Trends in expression of DEGs in susceptible mulberry at 17 days, in resistant mulberry at 23 days, after inoculation with nematodes (GBjz17d) (A), (KBjz23d) (B), 17 dpi (KBvsGB-GBjz17d) (C) and (KBvsGB-KBjz17d) (D) relative to expression in uninoculated (control) samples and the number of genes for each profile (B). The lower left corner of the trend block is the P-value. Smaller values indicate stronger enrichment (as shown in the graph below).

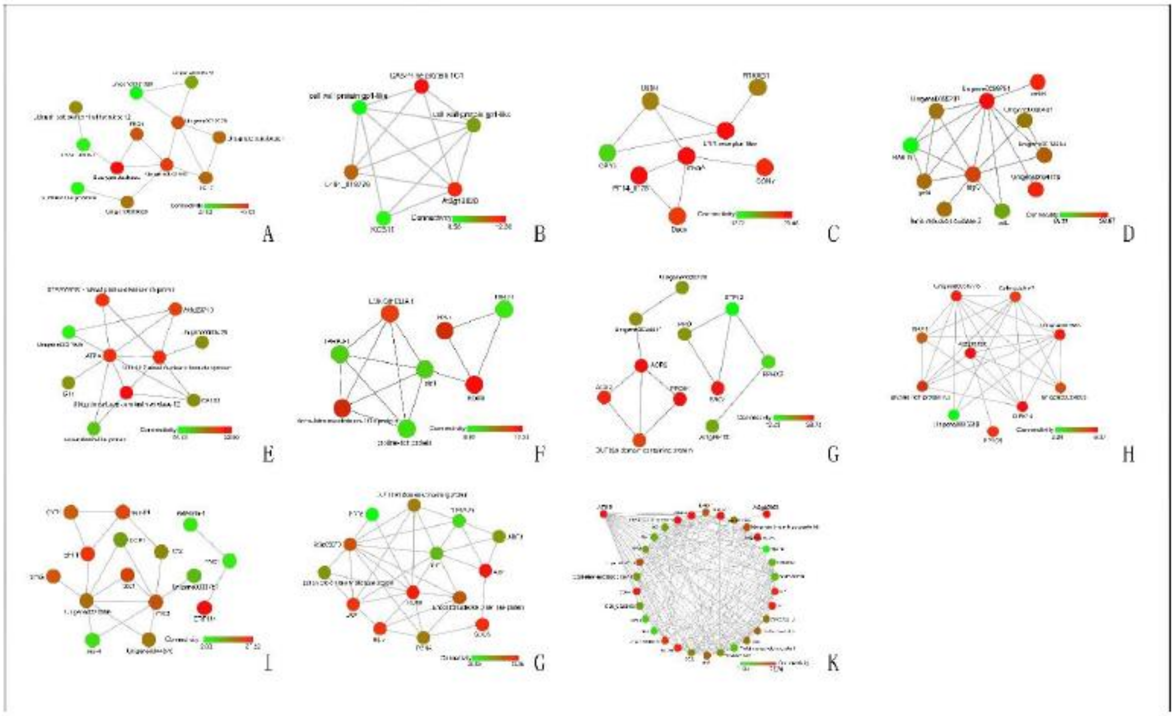

A: GBjz17d profile1 B: GBjz17d profile3 C: GBjz17d profile16 D: GBjz17d profile18
E: KBjz23d profile0 F: KBjz23d profile12 G: KBjz23d profile15 H: KBjz23d profile19
I: KBvsGB-KBjz17d profile3 J: KBvsGB-KBjz17d profile12 K: key trend of KBvsGB-GBjz17d

Fig.S2 Co-expression network analysis diagram of key trends
