# Supplementary material for: Transcriptome analysis of resistant and susceptible mulberry responses to Meloidogyne enterolobii infection
Source: BMC Plant Biol. 2021 Jul 16;21:338. doi: 10.1186/s12870-021-03128-w (PMC8285880; doi:10.1186/s12870-021-03128-w)
Supplement: Supplementary file 2 — Additional file 2: Table S1. qPCR reaction system. Table S2. Conditions for qPCR reaction. [file 12870_2021_3128_MOESM2_ESM.docx]

.

Table S1 qPCR reaction system

| Component | Volume |
| --- | --- |
| 2× SYBR Green Pro Taq HS Premix | 10 μL |
| Primer F (10 μM) | 0.4 μL |
| Primer R (10 μM) | 0.4 μL |
| Template | 2 μL |
| RNase-free water | up to 20 μL |

Table S2 Conditions for qPCR reaction

| Reaction stage | Temperature | Time | Cycles |
| --- | --- | --- | --- |
| Pre-denaturation | 95℃ | 30 s | 1 |
| Amplification | 95℃ | 5 s | 40 |
|  | 65℃^a^ | 30 s |  |
| Melting curve | 95℃ | 30 s | 1 |
|  | 65℃ | 30 s |  |
|  | 95℃^b^ | - |  |
| Condensation | 40℃ | 10 s | - |

^a^When setting the annealing temperature of the amplification stage to 65°C, ‘Single’ must be selected for the Acquisition Mode to ensure fluorescence signals will be collected during this period.

^b^Continuous must be selected for the acquisition mode of the second 95°C stage of the melting curve without setting the time.
